# Reconstitution and Transmission of Gut Microbiomes and Their Genes between Generations

**DOI:** 10.3390/microorganisms10010070

**Published:** 2021-12-30

**Authors:** Eugene Rosenberg, Ilana Zilber-Rosenberg

**Affiliations:** Department of Molecular Microbiology and Biotechnology, Tel Aviv University, Tel Aviv-Yafo 6997801, Israel; ilany43@gmail.com

**Keywords:** microbiome, holobiont, hologenome, microbiota transmission, gene/function transmission, vertical transmission, horizontal transmission

## Abstract

Microbiomes are transmitted between generations by a variety of different vertical and/or horizontal modes, including vegetative reproduction (vertical), via female germ cells (vertical), coprophagy and regurgitation (vertical and horizontal), physical contact starting at birth (vertical and horizontal), breast-feeding (vertical), and via the environment (horizontal). Analyses of vertical transmission can result in false negatives (failure to detect rare microbes) and false positives (strain variants). In humans, offspring receive most of their initial gut microbiota vertically from mothers during birth, via breast-feeding and close contact. Horizontal transmission is common in marine organisms and involves selectivity in determining which environmental microbes can colonize the organism’s microbiome. The following arguments are put forth concerning accurate microbial transmission: First, the transmission may be of functions, not necessarily of species; second, horizontal transmission may be as accurate as vertical transmission; third, detection techniques may fail to detect rare microbes; lastly, microbiomes develop and reach maturity with their hosts. In spite of the great variation in means of transmission discussed in this paper, microbiomes and their functions are transferred from one generation of holobionts to the next with fidelity. This provides a strong basis for each holobiont to be considered a unique biological entity and a level of selection in evolution, largely maintaining the uniqueness of the entity and conserving the species from one generation to the next.

## 1. Introduction

The hologenome concept of evolution [1,2] is based on four principles: (1) All animals and plants are holobionts, consisting of the host and diverse microbiota, including Bacteria, Archaea, eukaryotic microorganisms, and viruses. (2) Interactions between the host and its microbiota often affect the fitness of the holobiont in a beneficial manner, though negative interactions can also occur. (3) Microbiomes are transmitted between generations. (4) Alterations in either the host or the microbiome genomes (the hologenome) can lead to genetic variation and evolution. The microbiome genome is dynamic and changes more rapidly than the host genome. These principles stress the strong connection between the microbiome and the host in which a mutual adaptation has to evolve to achieve the robustness necessary for long-term survival [3,4]. Moreover, if these four principles are correct, then a holobiont with its hologenome is a level of selection in evolution [1,5]. In addition, and as a result, the approach to biological individuality and the concept of self has to undergo a change [6,7,8].

Out of the four principles, the main one that has been challenged is the transmission of microbiota between generations [9,10,11]. The critics argue that there is insufficient evidence to support the general claim that microbiomes are reconstructed each generation with fidelity. That seemed to us a good reason to focus on this intriguing subject and try to better understand its complexity.

It should be mentioned that several recent reviews have been published on different aspects of microbiome transmission. These reviews discuss plant microbiomes [12,13,14,15], animal microbiomes [4,16,17,18,19,20], and human microbiomes [21,22,23,24,25]. This article is an updated opinion review that discusses plants and animals, including humans.

In a nutshell, this article critically examines the evidence, pros and cons, for transmission of microbiota and their genes between holobiont generations. However, there are some obstacles that lie ahead, the main ones being the diverse routes by which microbiota are vertically and horizontally transmitted between hosts [26,27] and the limits of detection of rare microbiota within holobionts [28,29,30]. Additionally, although the number of studies on the microbiota of animals and plants is rapidly expanding [21,31,32], there is still a paucity of species in which transmission of microbiomes have been studied in depth [33,34,35], which makes it challenging to generalize. However, we shall try to come to some general conclusions.

## 2. Modes and Fidelity of Transmission

*As is well known, the gastrointestinal tract is sterile in the normal fetus up to the time of birth. During normal birth, however, the baby picks up microbes from the vagina and external genitalia of the mother and any other environmental source to which it is exposed*.

—Dwayne C. Savage [36]

Whereas animal and plant genomes are transmitted between generations by a well-established universal semi-conserved mechanism, microbiota and their genes are transmitted by a variety of different vertical, horizontal modes (Table 1), and as will be discussed later, also by mixed modes. Vertical transmission is defined as the movement of microbiota from parent to offspring without mixing with microbes in the environment, whereas horizontal transmission is defined as coming from the environment [5]. We shall first examine the phenomena of vertical and horizontal transmissions in different organisms and discuss the fidelity of the phenomena, then discuss aspects of quantitative assessment of transmission and finally we will derive some general conclusions from the data presented in the paper.

For a holobiont to be considered a level of selection in evolution, a significant fraction of its microbiome must be transferred between generations [5,8]. The diverse modes of transmission by different animals and plants make it difficult to derive generalizations concerning the fidelity of transmission. However, what is clear is that, in general, a functional microbiome has to be reconstituted each generation, thereby achieving fitness of the holobiont throughout its life. This conclusion leads to a number of major questions regarding the mode of transmission of the microbiome: What fraction of the microbiome is transferred vertically and what fraction horizontally or mixed? Does it matter if the microbiome is reconstituted by vertical and/or horizontal transmission? How important is the transmission of specific taxa as opposed to transferring specific and necessary functions/genes of the microbiome to the next generation? This section examines the fidelity of transmission of the different modes.

### 2.1. Vertical Transmission: Modes and Fidelity

Accurate vertical transmission and co-evolution have been demonstrated in many biological systems, co-evolution being defined as the process in which two or more species reciprocally affect each other’s evolution [37]. Funkhouser and Bordenstein [38] have argued that microbial maternal vertical transmission is widespread across animals. Vertical transmission is most important in assuring fidelity and accurate reconstructing of the next generation holobiont and hologenome.

#### 2.1.1. Vegetative Reproduction

Vegetative reproduction is an asexual reproduction occurring when no embryo is produced. Many plants and some animals can propagate asexually from somatic cells [39,40,41,42]. As a result of this type of reproduction, the microbiome is transferred vertically to offspring as part of the separated tissue. The most obvious example is vegetative reproduction in plants. Diverse plants can multiply vegetatively by producing runners, rhizomes, or root sprouts [43]. Although temporarily connected to the parent, if the segments can live independently after separation, it results in vegetative propagation [44]. Vegetative reproduction also occurs in some simple animals. The animal is broken, into two or more pieces; each fragment can form a fully-grown new organism, identical to the parent. For example, fragmentation occurs in corals [45], worms [46], sea stars [47], and sponges [48]. Budding is another type of asexual propagation in animals, resulting from the outgrowth of a part of the body leading to a separation of the bud from the original organism and formation of two individuals, one larger than the other. Budding occurs in sponges [49], sand dollars [50], *Hydra* [51], echinoderms [52], and bryozoans [53].

#### 2.1.2. Cytoplasmic Inheritance, Germ Cells, and Fetuses

Cytoplasmic inheritance, also referred to as extra nuclear inheritance, is transmission of genes that occur outside the nucleus. It is found in most eukaryotes and includes inheritance of mitochondria and chloroplasts, which can be considered “extreme symbionts” because though now considered organelles, they originated from alphaproteobacteria and cyanobacteria, respectively [54,55]. In general, all animals inherit their mitochondria only from their mother [56]. Maternal transmission of mitochondria is so precise that it can be used in tracing evolutionary lineages and population migrations [57]. Similarly, chloroplasts are maternally transmitted [58], with rare cases of paternal inheritance [59].

Vertical transmission from parent to offspring via oocytes occurs with several invertebrate endosymbionts, where the microorganisms are present in or on the reproductive cells. In the aphid–*Buchnera* symbiosis, for example, bacteria are intracellularly located in bacteriocytes and are transferred to and transmitted via the eggs [60,61,62]. Another well-studied example of vertical transmission via oocytes is in the *Drosophila*-*Wolbachia* endosymbiosis. *Wolbachia* is the most common endosymbiont in insects [63,64]. This endosymbiont is always present in female germ line stem cells, so that translocation of the symbiont is not necessary. It is well-established that endosymbionts in insects are vertically transmitted between generations with high fidelity and co-evolve with their hosts [65]. Some of these close associations between endosymbionts and their insect hosts are apparently evolutionarily stable for hundreds of millions of years [66,67], and during this long period a host–symbiont co-speciation has occurred [61]. Since these symbionts are unable to live outside of their host cells, it is unlikely that they are acquired from the environment, but rather, they are faithfully vertically transferred from mother to offspring [38]. In strictly vertically transmitted intracellular associations, mutation and genetic drift over millions of years has resulted in severe genome erosion of the symbiont [68].

In many corals, the endo-symbiotic algae, *Symbiodinium*, are transmitted directly from parent to offspring via eggs. This mechanism of symbiont transmission perpetuates *Symbiodinium* diversity found in the parent through multiple generations [69]. In some sponges, symbiotic bacteria penetrate into growing oocytes by endocytosis [70]. Many insects such as fruit flies [71], stinkbugs [72], and other arthropods [73], transfer their microbiota from their gut to the eggs they lay.

Inside the shells of chicken eggs exist an abundant and highly diverse bacterial population, derived from the mother and associated with the developing chick fetus [74,75,76]. It appears that the chicken embryo obtains its intestinal microbiota from the egg white prior to hatching. Thus, microbiota is transmitted vertically from hen to offspring via eggs. Eggs of wild birds and lizards also contain in ovo microbial communities, presumably through the inoculation of egg yolk prior to shelling [77].

The presence of bacteria inside eggs raises the question of whether other animals also transfer microbiota directly to their fetuses prior to birth. In humans, it was accepted for more than a century, that the fetus matures in a bacteria-free environment prior to birth. However, several publications during the last ten years report the presence of bacteria in the human fetal environment including inside the placenta [78,79,80,81,82,83]. It has also been reported that calf fetuses are not sterile, but are spatially colonized before birth by a pioneer microbiome [84].

A number of scientists have challenged the prenatal microbiome hypothesis, identifying contamination as a major issue [85,86,87,88]. Furthermore, the fact that it is possible to generate germ-free mammals by Caesarean section suggests that the fetus must be sterile. A panel of microbiome experts, who have not been directly involved in the debate, were asked to discuss these issues and publish their thoughts on the controversy [89]. The following statement by Martin Blaser reflects the general conclusion of the panel: “Any claim that there is indeed an indigenous microbiota would need to be well-substantiated and unequivocal, since it would need to surmount both existing theory and logic. At this point, no finding has passed that threshold, in my opinion.”

Plant seeds play an important role in the vertical transmission of bacteria and fungi between successive generations, thereby ensuring their presence in the next generation [90,91,92]. Seeds have the potential to remain in a dormant state for a considerable period of time until growth conditions become suitable for their germination and development into a new plant [93].

#### 2.1.3. Coprophagy

Coprophagy is feces-eating behavior [94]. This mode of microbial transmission via feces consumption has been reported in a large number of animal species, including insects, rodents, piglets, foals, dogs, and nonhuman primates [95]. Coprophagy allows the young animals to obtain gut symbiotic microbes, which they require to digest complex polysaccharides, from their parents [95,96,97]. Koalas use a special adaptation of coprophagy [98,99]. For months, the joey relies entirely on the mothers’ milk; subsequently, the mother produces a special type of feces (pap), which the joey consumes over several days. This process facilitates vertical transmission of microorganisms that are able to digest eucalyptus leaves. Other examples of direct transmission via feeding feces is observed in termites [100,101], and some cockroach species [102,103], in addition to some insects that lay eggs in their feces that is consumed by the hatching offspring [104].

Coprophagy can bring about also a mixed mode of transmission (MMT, see further in this review), namely, not just strictly vertical, but also partially horizontal, as is common among some insects [105]. This takes place when the offspring consume feces from the ground, not directly from the parent.

#### 2.1.4. Regurgitation of Food

Many bird species regurgitate food into the mouths of their young, thereby ensuring a direct vertical mode of microbial transmission [106]. Regurgitation and ingestion of food by primates are behaviors that are pervasive in zoos, but have not been observed in animals in the wild [107,108]. Some beetles feed regurgitated food, and potentially also bacteria, to larvae throughout their development [109].

Just as mammals breastfeed their offspring, parent rock pigeons regurgitate pigeon milk from an enlarged part of their esophagus to feed the chicks, termed squabs [110]. However, unlike mammals, both male and female pigeons produce pigeon milk [111]. The relatively high percentage of shared bacterial species between squabs and their parents is a strong indication that the squabs obtain bacteria through regurgitation of parental milk [112,113].

#### 2.1.5. Physical Contact

The mode of delivery in all animals shapes the acquisition and structure of the initial microbiome in newborns [114,115]. The newborn human gut is initially colonized via inoculation with maternal vaginal and fecal microbes, when the baby passes through the birth canal (vertical transmission). Direct vertical transmission during birth has been demonstrated in several animals, including bears [116], great apes [117], bats [118], mice [26], beetles [119], and fishes [120,121], in addition to humans [122,123,124]. Moreover, Li et al. [124] demonstrated that antibiotic administration to mothers during birth reduced considerably and changed the vertically transmitted species.

Cesarean section (CS) delivery is one of the strongest disrupting factors of the normal colonization process and has been reported as a risk factor for disorders in later life [125]. Human infants born by CS are colonized initially and primarily by skin microbiota [126,127]. Subsequent colonization occurs in humans by close physical contact of the offspring with parent, family, and community members [128]. Kissing, hugging, and touching result in the transfer of microbes [129]. Kort et al. [130] demonstrated that, in humans, an average 10-s open-mouth kiss transfers approximately 80 million bacteria. However, social transmission, between individuals, within herds and between populations, all being horizontal, also occurs in many animals [131], including non-human primates [132], herbivores [133], and birds [134].

In humans, an early discovered example of a microbe being transmitted from parent to offspring for many generations came from a detailed analysis of the sequence diversity of DNA isolated from specific strains of *Helicobacter pylori,* present in different geographic human populations [135,136]. *H. pylori* is acquired early in life from mothers and occasionally from family members [137,138]. The fact that a distinct strain-specific sequence remains for centuries in offspring of an individual that has migrated to a different geographical location, argues for accurate transmission. This has led to the use of *H. pylori* in discovering details of human migration at individual and population levels [139,140].

One way to assess whether microbes are influenced by the genetic composition of the host, and therefore conserved across generations, is by measuring heritability (*h*^2^)—the proportion of phenotypic variance explained by genetic variance [141]. For example, recently it was reported that 97% of microbiome phenotypes in wild baboons were significantly heritable [142]. In humans, because the overall similarity of gut bacterial community composition between adult mono- and di-zygotic twin pairs is largely the same, it is reasonable to conclude that physical contact during and after birth has a greater influence as compared to genetic relatedness, in determining gut microbial composition [143,144]. Although it is not clear how much of the human gut microbiome is transferred from mother to infant, several groups of bacteria, such as specific strains of *Bifidobacterium breve* and *Lactobacillus plantarum,* were shown to be always present in the infant gut and their mothers’ feces and milk [145,146,147], providing direct support for accurate vertical transmission of these strains. Strains from the classes Actinobacteria and Bacteroidia, which are essential components of the infant microbiome, have been shown to be vertically transmitted from mother to offspring and persist for at least 1 year after birth [148].

#### 2.1.6. Breastfeeding

Breastfeeding is another important route of maternal vertical microbial transmission to offspring in mammals [149,150]. Breast milk from healthy human mothers contains ca. 10^5^ bacteria per ml, composed of hundreds of species of commensal and mutualistic bacteria [151]. These bacteria are provided to the newborn infants’ gastrointestinal tract during early and critical periods of development [152]. Analyses of the DNA of several bacterial strains isolated from mothers’ milk demonstrated that they were identical to those found in the offspring [153]. Asnicar et al. [154] demonstrated that several specific strains (e.g., *Bifidobacterium bifidum*, *Coprococcus comes* and *Ruminococcus bromii*) were present in samples from the same mother–infant pair, while being clearly distinct from those carried by other pairs, which is indicative of vertical transmission.

One particular bacterial genus in breast milk that warrants special attention is *Bifidobacterium*. Members of this genus are uniquely genetically adapted to utilize glycans present in human milk as an energy source [155]. While this genus makes up only a small percentage of human milk bacteria, it dominates the gastrointestinal microbiome of breastfed infants [156], where it plays important roles in shaping the infant GI microbiome and programming health [157]. The clear demonstration that these beneficial bacteria co-evolved with humans can be perceived from their interaction with human milk oligosaccharides (HMOs). These HMOs, which are complex carbohydrates and uniquely abundant in breast milk, have evolved while supporting the assembly of a healthy gut microbiome in the human infant [158]. The HMOs do not provide energy to the infant; instead, they are used exclusively by the gut microbiota that have evolved specific enzymes that metabolize HMOs [159]. These data demonstrate a clear co-evolution of certain strains of bacteria with humans [37].

Fungi and viruses have also been detected in the breastmilk of healthy human mothers, as well as the milk of other animals [160,161,162]. A thorough review on milk microbiomes has recently been published by Oikonomou et al. [152].

The origin of human milk microbes is not clear [163]. One suggestion is that a preexisting mammary gland microbiome acts as the initial seed for the human milk microbiome [164]. Another possibility is that microbes access the mammary tissue in non-pregnant, non-lactating individuals because mammary glands are exposed to the environment via the nipple. In fact, bacterial DNA has been identified in non-lactating mammary tissue from women undergoing breast surgery [165]. The non-lactating mammary tissue of nonhuman primates has also been shown to contain bacterial DNA [166]. Thus, bacterial DNA may be present in the mammary gland prior to the onset of first lactation. These bacterial DNA profiles share some features with the human milk microbiome. Furthermore, it was possible to culture bacteria from breast tissue suggesting the presence of a viable mammary microbiome [167,168]. A third possible rout is suggested by studies demonstrating that bacteria consumed orally by lactating women or animals can subsequently be isolated from their milk [169], indicating that bacteria may also reach the mammary gland through an internal pathway. The proposed entero-mammary pathway involves immune cell-mediated bacterial translocation from the mother’s gastrointestinal tract into the mammary gland [170,171]. Bacterial translocation increases toward the end of pregnancy, possibly acting as a second seeding event [172]. Once the milk starts to be produced in the breast, the composition of the microbiome appears to change, probably due to the introduction of different bacterial substrates and immune factors present in colostrum and milk.

#### 2.1.7. Vertical Transmission over Evolutionary Time-Scales

Vertical transmission of bacteria over evolutionary time-scales was investigated by analyzing and comparing the 16S rRNA gene sequences of bacteria present in the great apes, including humans [173,174]. Since the host species phylogenies based on the composition of these bacterial communities was parallel to the evolutionary relationships of their hosts, Ochman et al. [173] concluded that “over evolutionary timescales, the composition of the gut microbiota among great ape species is phylogenetically conserved and has diverged in a manner consistent with vertical inheritance”. This conclusion was challenged by Moran and Sloan [9]. They correctly pointed out that vertical transmission of bacterial species, based on 16S rRNA gene sequences alone, cannot be used to prove co-evolution because it is possible, even likely, that over evolutionary timescales other strains of the same species (>97% identity in 16S rRNA gene sequence) could have been acquired from the environment (horizontally).

To overcome the problem raised by Moran and Sloan [9], Sanders et al. [175] developed the beta-diversity clustering method, which distinguishes between shared evolutionary history and environmental filtering. Applying this method to the great ape data led to the conclusion that great apes acquire microbiota largely from the environment, but retain a significant proportion of vertically transmitted microbes over long timescales. When the method was applied to turtle ants, the data indicated that vertical transmission of the entire bacterial community played an important role in the evolution and maintenance of the turtle ant/microbiome association [175].

Moeller et al. [176] used a different approach to test the fidelity of microbiota transmission in great apes. They employed rapidly evolving gene sequences instead of stable 16S rRNA genes, to analyze the fidelity of gut bacterial transmission in humans, wild chimpanzees, and wild bonobos. The analyses led to the conclusion that strains of the common gut bacteria, Bacteroidaceae and Bifidobacteriaceae, have been preserved within host lineages across hundreds of thousands of host generations. Since the divergence times of these co-speciation gut bacteria are congruent with those of hominids, it can be concluded that nuclear, mitochondrial, and gut bacterial genomes, i.e., hologenomes, diversified in concert during hominid evolution. Thus, though a predominant fraction of gut bacteria originates from the environment, a significant fraction has co-evolved for millions of years with hominids and participated in their adaptation and development [177]. Furthermore, Rampelli et al. [178] demonstrated that in the gut microbiota of Neanderthal occupational deposits, dating back 50 thousand years, many well-known beneficial gut commensals already populated the gut microbiome of *Homos* as far back as the last common ancestor between humans and Neanderthals.

### 2.2. Horizontal Transmission

Most symbiotic microorganisms, being part of a holobiont, are adapted to living within their host; moreover, some cannot replicate outside, which reflects their adaptation to specific niches in their hosts [179,180]. In humans and in mice, the two most abundant bacterial phyla are the Firmicutes and the Bacteroidetes, most of which do not grow outside of their host [181]. Adaptation to growth in their hosts is not only common in animals, but is also common in plants [182]. A consequence of this is that symbiotic microbes can generally outcompete environmental organisms for residence in their hosts.

Regardless of this competition mechanism, infection of holobionts by environmental non-pathogenic (also pathogenic, but they are not the subject of this article) microbes occurs all the time. However, in order for such environmental bacteria to persist and multiply in a host they have to be well adapted to the host or else the immune system and the resident microbiota will not enable their colonization, and second, they have also probably to participate in some way in the fitness of the holobiont or at least be commensal.

Just as there are multiple modes of vertical transmission, so can horizontal transmission be divided by the source of the acquired microbe, though it is more difficult to prove its origin. Offspring often acquire horizontally transmitted microbes not from a parent, but from a family member or any other organism in which they are in close contact with. For example, it has been reported that humans acquire microbes also from their pets [183,184]. Although in some cases horizontal transmission is apparently the main mode of transmission, mostly it takes place together with vertical transmission (see discussion further in the paper, mixed mode).

One of the arguments against considering the holobiont with its hologenome as a level of selection in evolution is the pervasiveness of horizontal transmission, which is claimed to prevent reliable reconstitution of the microbiome between generations [10,185,186]. Douglas and Werren [10] have argued that host-symbiont partner fidelity is weak for many horizontally acquired symbioses. As examples, they note that gut microbiota in genetically defined strains of laboratory mice and *Drosophila melanogaster* varies among laboratories, even within one laboratory, over time [187,188,189,190]. In our opinion, these examples may indicate that the relative numbers of specific microbes in the microbiome can change as a function of environmental factors and when the frequency of a species or strain goes below a critical number it can no longer be detected, as will be discussed in Section 4. An additional study of eight marine sponges, spanning two classes, casts doubt on the consistency and faithfulness of transmission of microbiomes between generations [11]. The data from the study suggest that siblings receive only a small set of identical symbionts and that the majority of these microbes originate from the seawater where they were probably selectively acquired by the adult parent before being vertically transmitted to offspring. The authors conclude that it is unlikely that microbiota have co-evolved with particular sponge species. However, the authors are aware of the finding that adult sponges have highly species-specific microbiomes [191] and suggest two possible explanations for the lack of vertical transmission of parental microbes to larvae. First, they suggest, as others do [192] that priority influences community, namely, only a small number of essential microbes need to be transferred vertically from parent in order to build eventually a functional adult microbiome and holobiont. Second, though sponges filter an enormous quantity of water with a vast numbers of microorganisms (mostly as food) they are able to recognize specific microorganisms via their innate immune system and specific molecular structures. Both these mechanisms point to the process of development of the mature microbiome that becomes species specific. Ramos et al. [193] discussing functional (not taxa) microbial composition across generations (see further in this article) also suggest that in humans vertically transmitted microbiota begin a predictable change of functions whose fitness depends on the arrival of additional bacteria.

Despite the evidence for weak vertical transmission and higher horizontal transmission in different organisms and systems, there exist examples of faithful reconstructions of a holobionts and hologenomes by horizontally transferred symbionts [194,195,196,197,198,199,200].

Probably the best-studied example of environmental transmission occurs in the squid-*Vibrio fischeri* symbiosis [195,201]. The female squid lays clutches of hundreds of fertilized eggs, which hatch almost synchronously at dusk. In parallel, adult squids release large amounts of *V. fischeri* into the water at dawn every day. The growing embryos develop an immature light organ that harbors pores leading to separate epithial-lined crypts. These crypts become colonized by the released *V. fischeri* from the surrounding water. Accurate transmission is ensured by the developing squid that provides a niche only for the *V. fischeri* that emits light and is able to maintain a stable association. Thus, the squid microbiome is reconstituted every generation by a specific environmental transmission, though not necessarily from their specific parent.

Horizontal transmission in the marine environment is often mediated through a mucous interface and requires complex recognition mechanisms, most often involving sugar-lectin interactions and cellular surface structures that select specific symbionts from the environment and avoid pathogen invasions [202].

Horizontal transmission among many land animals, occurring via consumption of plant material, is probably part of the natural transmission process necessary for passage of microorganisms, that breakdown plant material, to offspring. Studies have demonstrated that bacteria associated with raw-eaten leafy green vegetables, or even with processed foods, are ingested by their consumers, in other words they are horizontally transferred to herbivore and omnivore animals, including to humans [203].

An interesting recent publication describes horizontal transmission of gut microbiota between two different animal species living in the Tibetan Plateau, the pika (a small hare-like mammal) and the yak [204]. The pika and yak have been sharing the same habitats, and consuming similar foods for more than a million years and therefore are considered competitors. Surprisingly, it was observed that when the yak population increased, causing overgrazing, the population size of pikas also increased. When trying to discover the underlying mechanism for this unexpected finding and examining the microbiomes of sympatric and allopatric pikas and yaks, the researchers discovered that sympatry increased both gut microbial diversity and similarity between pikas and yaks. In sympatry, pikas acquired 2692 OTUs from yaks, and yaks obtained 453 OTUs from pikas. In the pikas, these horizontally transmitted bacteria enhanced the enrichment of pathways related to prebiotics and immunity. In yaks, the horizontally transmitted bacteria enhanced pathways related to hepato-protection, xenobiotic biodegradation, and detoxification. Thus, pikas and yaks may not be chiefly competitors, but rather their relationship may be characterized by reciprocity through the horizontal transmission of gut microbiota. The mode of transmission probably occurs by pikas eating the feces of yaks [205], and yaks acquiring pika excretions in the soil [206].

Horizontal transmission has been shown to exist in many additional animals and plants. Studies in both humans [207] and non-human primates [208] provide strong evidence for the contribution of horizontal transmission to microbiome assembly. Tung et al. [209] showed that social networks and social interactions in wild baboons, could predict microbiome structure, even after controlling for shared environment, diet, and relatedness. In another study, it was reported that in several different surveyed non-human primate species microbiomes varied with host species, but importantly also by social groups within species [210]. Other examples of horizontal transmission include plant to other plants via fungal propagules (a vegetative structure that can become detached from a plant) in grasses [196], via the nesting environment in wild birds [211], and from the surrounding water in fish [212,213]. Horizontal transmission of endosymbionts of insects such as *Wolbachia, Ricketssia*, *Cardinium* and the parasite of a leafhopper via plants has also been described [214].

Recently, it was reported that microbes could be transmitted not only from plants to animals, but also from animals to plants. Lettuce grown in soil containing cattle manure, acquired in their leaves manure-borne microbes that include antibiotic resistance genes, which may assist in protection against infection [215]. Moreover, antibiotic resistance genes, acquired from poultry litter was present in the roots of lettuce [216].

Roughgarden [217] has presented a mathematical model to examine how holobiont selection might operate and to assess its plausibility as an evolutionary force. In one variant of the model, offspring obtain microbiomes from their parents directly (vertical transmission). In the complimentary variant, microbiomes of offspring are assembled from source pools containing combined microbiomes existing in the near environment from all parents, as in the squid/*Vibrio* system (horizontal transmission). According to both variants of the model, holobiont selection causes evolutionary change in holobiont traits. Therefore, holobiont selection is plausibly an effective evolutionary force with either mode of microbiome transmission.

What are the advantages of horizontal transmission of microbiota? Since some of the microbiome members are not vertically transmitted in a reliable way [218], strong natural selection would favor hosts that can seize useful symbionts from the local environment [219,220]. Another advantage of acquiring microbes from the environment is the possibility of genetic variation and evolution of holobionts by the occasional acquisition of a novel beneficial strain [221]. The presence of an environmental component implies that offspring can be colonized by beneficial symbionts as well as environmental bacteria that can harm the host [105,222]. Accordingly, the establishment of a healthy microbiome will depend on the ability of the holobiont to acquire beneficial bacteria and exclude pathogens [223]. During microbiota transmission (whether vertical or horizontal), selection by the host and/or by components of the microbiome, is a key process in establishing and maintaining a holobiont microbial community that is fit for the specific host in its environment [224].

### 2.3. Mixed Vertical and Horizontal Transmission

Symbiont transmission modes are best conceptualized on a spectrum between strict vertical and strict horizontal transmission. In between these extreme modes, exist the mixed mode of transmission (MMT), involving vertically and horizontally transmissions of the same microbe or microbiome or host switching of the same microbe [225]. Ebert [225] claimed that MMT is the dominant mode of transmission in the animal and plant worlds. A systematic review and meta-analysis of modes of microbiota transmission performed by Russell [226] showed that out of a total of 528 analyzed symbioses, 43% were strictly vertically transmitted, 21% were strictly horizontally transmitted, and 36% exhibited some form of MMT, which he predicted to be generally underestimated because of relatively fewer data. In general, Russell concluded that external modes of vertical transmission (e.g., secretions), as opposed to internal modes (e.g., intracellular), predisposed holobionts to mixed modes of transmission. In addition, Russell observed that a higher frequency of vertical transmission existed on land and a higher frequency of horizontal transmission existed in aquatic environments [226,227]. The observation of horizontal transmission being more abundant in the marine than in terrestrial environment could arise from the simple fact that water is a conducive medium in which desiccation and osmolarity do not represent a problem, thus encouraging horizontal transmission and host to-host transfer events [4]. For example, in the sponge, *Plakina cyanorosea*, harboring a relatively low microbial abundance, transmission of its microbiome relies primarily, but not exclusively, on horizontal transmission [220]. However, there is now also evidence for maternal vertical inheritance in deep-sea animals as well [220,228,229], indicating a mixed vertical and horizontal transmission. In chemosynthetic symbionts in deep-sea mussels both horizontal [230] and vertical transmission [231] have been reported. Transmission of chemosynthetic symbionts in these organisms is an important determinant of population structure and genome evolution [230]. It seems that stochastic effects on the colonization of horizontally transmitted bacteria may manifest themselves also on land in differences of microbiota strain composition among hosts, as was reported for the nematode *Caenorhabditis elegans* [232].

Moeller et al. [26] working with two strains of mice from the wild, that were bred for up to 11 generations in the lab, demonstrated an individual and population level transmission which was predominantly vertical, but also environmental, namely a mixed transfer of microbes. They also observed that the mode of transmission tends to be dependent on bacterial genera. In addition, aerobic bacteria tended to be transmitted horizontally and anaerobic bacteria-vertically. Other examples of mixed transmission of microbiomes were observed in great apes, as discussed above [175], in ruminants [233,234], in mammalian herbivores [235] in insects [199] and in corals [69].

## 3. The Core Microbiome and Transmission of Functions and Genes

The most accepted definition of a core human microbiome is a group of microbial taxa or genes that are shared by all or most humans [236,237,238]. This definition can be applied to any other animal or plant holobiont. The reported data to date have led to the conclusion that a universal taxonomic organismal core is small [239,240]. However, it can be argued that it may be much larger than currently recognized because of the failure to detect rare microbes, as will be discussed in Section 4, and the rare undetected microbes may have crucial functions when conditions change [241].

In addition to the taxonomic aspect of the core, the definition of “microbiome core” includes also the element of common genes, which implies common functions. A large volume of research during the last twenty years has demonstrated that microbial genes are responsible for many functions essential for the fitness of animal and plant holobionts (reviewed by [2,238,242,243]). These essential functions must be and are, in fact, transmitted between generations [123]. In principle, the microbial genes coding for these functions, not necessarily the taxa, could be transferred vertically or acquired from the environment by a variety of mechanisms, as discussed in this review. As Taxis et al. [244] wrote, “The players may change but the game remains”, and Doolittle and Booth [245] wrote “it’s the song not the singer.” The same functional gene or isogene can be present in different bacteria in the microbiome, i.e., there is gene redundancy in the microbiome [246,247]. Thus, if a particular bacterium possessing an essential functional gene decreases in abundance or is lost, the function may be provided by genes of other bacteria.

Ramos et al. [193] looking for experimental data to support the hypothesis of “It’s the song not the singer” tested a number of variables: The first was transmission of functions between generations in databases on zooplankton, mosquito, and plants; the second was the change of functional microbiota with time, up to three years, using a human database; the third was simulation of microbiota communities to test if pairwise interactions lead to stable community compositions. Although the results did not demonstrate transmission of function, they suggested that “the vertically transmitted microbiota starts a predictable change of functions performed by the microbiota over time, whose robustness depends on the arrival of diverse migrants. This succession culminates in a stable functional composition state.” They concluded that if their proposed mechanism proves to be well founded for different hosts, it would support the concept of holobionts acting as units of selection in evolution.

Suárez [248] argued that the holobiont/hologenome can act as a genuine level of selection both in the form of an interactor and in the form of a reproducer. To do so, he maintains that the microbiome should be characterized in functional rather than taxonomic terms.

## 4. Quantitative Assessment of Transmission of Microbiota

Reliable and meaningful scientific conclusions from microbiome studies rely on accurate analysis of microbial communities [249]. Several recent publications have discussed procedures for overcoming threats to reproducibility, replicability, robustness, and generalizability in microbiome research [250,251,252,253,254]. In order to evaluate if any particular microbe is transmitted between generations, it is essential to characterize it at the strain level. It is also necessary to detect rare species.

### 4.1. False Positives

With regard to this article, a false positive refers to an incorrect claim that a specific microbe is transmitted from parent to offspring. The vast majority of studies comparing microbiota in parents and offspring has relied on 16S rRNA gene determination, using > 97% identity to characterize a bacterial species [255]. As many scientists have pointed out > 97% identity of 16S rRNA gene sequences does not prove that two bacteria are identical [256,257,258]. This is particularly relevant to transmission studies because this methodology cannot determine if the offspring obtained the bacterium vertically from the parent [9]. A different bacterium with >97% identity of 16S rRNA gene sequences could have been acquired from the environment harboring slightly different genes. To overcome this problem, strain-specific gene sequences, although still largely underrepresented in public repositories, can be employed to identify bacteria down to the strain level [259,260]. Although not always possible, the most decisive method to prove that a particular bacterium was transmitted from parent to offspring is to perform whole genome sequencing of the bacterium isolated from parent and from offspring [261].

Another source of false positives is bacterial or DNA contamination in reagents used for DNA extraction and PCR [262,263]. Maqsood et al. [264] reported that in his study several infant stool bacterial microbiomes were indistinguishable from buffer background, and thus cannot be attributed to maternal transmission. In a different study, 54% of bacterial signals in the brain was explained by exogenous DNA contamination, and were thus false-positives [265]. DNase treatment has been suggested as a method for removing DNA contamination in PCR reagents [263].

### 4.2. False Negatives

The failure to find a particular bacterium in the microbiome does not mean it is not there. The more accurate conclusion is that it is not present at the limits of detection used in the experiment. As things stand to date, if a microbial species is present, but rare, it is often not detected [266]. For example, if a particular bacterial species was present in 10^3^ copies in the human colon (total bacteria = 10^13^–10^14^), it would be necessary to sequence 10^10^–10^11^ 16S rRNA genes to detect it. This is clearly not practical with the existing technology. In fact, statistical analyses indicate that the reported values of bacterial diversity in microbiomes are underestimations [267]. It is important to point out that even a single bacterium has the potential to amplify in numbers when conditions change to the benefit this specific bacterium, and thus may allow the holobiont to evolve by being more adapted to the new condition.

Recent research on the gut microbiota has largely been driven by the advent of modern sequence-based techniques [268,269]. Although these are powerful and valuable tools, they have limitations. For example, profiling gut microbiomes by both 16S rRNA gene sequencing and shotgun metagenomic sequencing techniques demonstrated several genera that are missed or underrepresented by each profiling method separately [270].

Another possible source of false negatives are spores. At least 50% of the bacterial genera from the intestinal microbiota of a healthy individual produce resilient spores, specialized for host-to-host transmission [271,272]. However, a phylogenetic assessment of the microbial communities has revealed surprisingly few spore formers [273], probably because spores are known to withstand many traditional methods of DNA isolation and are thus potentially undetectable [274]. The failure to detect rare bacteria and spores makes it difficult to prove or disprove modes of transmission.

## 5. Concluding Remarks

In this article, we discussed the importance of vertical and horizontal transmissions of microbes or functions, in the process of creating a holobiont with its hologenome as a unit of selection in evolution. In general, modes of microbiota transmission are diverse, and probably have evolved to fit the needs and the physical and physiological characteristics of the specific holobiont species.

Summing up the information discussed here, we suggest that the microbiome of holobionts, animals or plants, develops in parallel with the development of the host, eventually creating the mature holobiont. The initial microbiome of the offspring originates usually vertically from the parents, in many cases from the mother, and it has to include the microorganisms that are essential for its development. As the offspring develops, it collects additional microorganisms, which can be foreign, familial, or communal [275]. As was suggested by Fukami [192], Thomas et al. [191], Björk et al. [11], and Ramos et al. [193], eventually the adult microbiome reaches an equilibrium that involves the microbes and functions that are necessary for the survival of the adult holobiont under different conditions. This developmental process is in contrast to the genetic potential of the host that is available from the start.

The microbiome is comprised of a core that is species-specific and includes the essential functions for each developmental stage and environmental condition in addition to diverse microbes that characterize the individual holobiont. The size of the core is still not clear and neither is the question of what forms the core taxa or function, or both. At each point of time, in which the microbiome will be tested for taxa, a certain picture will emerge. This picture will reflect both the core and other individually specific microbes in the particular conditions (diet, health, temperature, etc.,) at the time of sampling. Thus, the picture that emerges, using the techniques available today, does not necessarily reflect the complete potential of microbes of the holobiont. It mirrors the microbes that are abundant (amplified) at the time of sampling, while other microbes that have decreased considerably in number because they are less fit for the present conditions will not be detected. It is important to note that although the latter may not be detected, they remain potential players when conditions change. However, while the full microbiome taxa may not be reflected in any specific time, genes from different taxa may fulfill the necessary functions needed for core and other activities.

There are two main advantages for vertical transmission. First, it ensures that the offspring acquires the essential genes that are necessary for the holobiont’s fitness and survival, at least at the initial stages, and it acts as a nucleus for construction of the adult microbiome. This is not left to chance. Second, vertically transferred microbes are adapted to life in their hosts (temperature, pH, immune system, etc.), while microbes acquired from the environment may not be as well adapted. In humans, who are the best studied holobiont, strain-level analyses reveal that offspring acquire a large fraction of their microbiota from their parents (mainly, the mother), suggesting vertical transmission of microbiota primarily from the maternal gut, but also from the vagina, skin, mouth, and breast milk [25,276]. It has often been reported that gut microbiota in early life is characterized by rapid and large changes in microbial diversity [277,278,279]. Microbiota that are absent from infants but present in childhood and adults are assumed to be acquired by horizontal transmission, especially from family members [128,280]. However, it is possible, even likely, that the bacteria were present in the infant, but below the detection limit and then amplified when conditions changed, especially nutrition. If this hypothesis is correct, there may be more vertical transmission and possible co-evolution than previously considered.

Let us now consider horizontal transmission. As suggested above, in order for a horizontally acquired microorganism to multiply in a host it must be accepted by the host and by the other members of the microbiome and in some cases it may also contribute to the fitness of the holobiont and even be obligatory to its existence. In spite of horizontal transmission being seemingly less accurate than vertical transmission in constructing the holobiont, the squid-*Vibrio* example may not be a special case, and we may find that horizontal transmissions are more common and obligatory than we can prove at this point of microbiome research. Holobionts many times live within the close environment of their own species—A community [275]. Thus, it seems logical to assume that as long as the holobiont achieves its potential necessary array of microbial functions to fulfill its needs during different life phases and conditions, and create a holobiont with an adult hologenome, it may be of small consequence if the functions are obtained by horizontal or by vertical transmission. To prove the point of the preciseness of core microbiome duplication from one generation, it will be necessary to compare accurately adult microbiomes by deep genome sequencing that will reach the level of even single microbes and by gene or function analysis.

Horizontal transmission has a definite advantage, supplying functions that were not present in the initial vertically transferred stock of microorganisms. It may insert necessary functions for the present and can bring new functions that may be necessary for the near and far future when conditions change, also participating in stable genetic variations and species evolution. The large variations in modes of transmission have an interesting implication: individuals can acquire and transfer symbionts throughout their lives, and not just during their reproductive phase. Furthermore, this flexibility implies that the environment can have an influence on the composition of the hologenome, on the one hand bringing in different microbes with the same functions, and on the other, bringing in new genetic material, assisting in short and long-term adaptation and evolution.

Let us sum up the arguments put forth regarding accurate microbial transmission and their support of the hologenome concept. First, the transmission may be of functions, not necessarily of species; second, horizontal transmission may be as accurate as vertical transmission; third, detection techniques may be limiting; and lastly, microbiomes develop with their host reaching maturity at adulthood. In addition, it should be born in mind that transfer of genetic material of the host from one generation to the next is also not always completely accurate, which is one of the mechanisms conferring uniqueness to offspring and possibilities of variation and evolution, regardless of a large common genetic denominator.

It is clear that despite the great variation in holobionts and in means of transmission discussed in this review, microbiotas and their functions are, in fact, transferred from one generation of holobionts to the next with fidelity. Such reliable transmission provides a strong basis for each holobiont to be considered a unique biological entity and a level of selection in evolution [2,5], largely maintaining the uniqueness of the entity and conserving the holobiont species from one generation to the next, in addition to contributing to adaptation and evolution of the holobiont [281].

## Figures and Tables

**Table 1:** Modes of transmission of microbiota.

Mode of Transmission	Examples
Vegetative reproduction (vertical)	Plants, worms, corals, sponges, sand dollars, bryozoans, starfish, sea urchins, sea cucumbers
Female germ cells: eggs, embryos and seeds (vertical)	Mitochondria, chloroplasts, aphid/*Buchnera*, *Drosophila*/*Wolbachia*, chicken embryo/microbiota, plant seeds/microbiota
Coprophagy (vertical & horizontal)	Insects, rodents, iguanas, rabbits, pigs, horses, elephants, pandas, koalas, primates, termites
Regurgitation of food (vertical)	Birds, beetles
Physical contact starting at birth (vertical & horizontal)	Most organisms
Mother’s milk (vertical)	Mammals
Environmental (horizontal)	Squid/*Vibrio fischeri*, grasses/endophytes, fish/microbiota
